# Piloting a New Curriculum: Guided At-Home Pediatric Regional Anesthesia Education Using a Portable Ultrasound

**DOI:** 10.7759/cureus.17933

**Published:** 2021-09-13

**Authors:** Michael R Greenberg, Marley B Lawrence, Fei Chen, Elizabeth M Ross

**Affiliations:** 1 Department of Anesthesiology, University of North Carolina at Chapel Hill School of Medicine, Chapel Hill, USA

**Keywords:** regional anesthesia, pediatric anesthesiology, education, virtual workshop, covid-19

## Abstract

Ultrasound-guided regional anesthesia is the standard of care for most regional blocks in pediatric anesthesiology.Training programs must educate physicians to perform regional blocks safely and efficiently. Hands-on learning with simulation and live models is the gold standard. The coronavirus disease 2019 (COVID-19) pandemic has greatly hampered our ability to safely hold in-person workshops. We describe an at-home, guided virtual workshop using portable ultrasound to safely continue experiential trainee education.

The primary objective of this pilot virtual workshop was to develop an effective experiential learning program without the need for live child models. The main goal was to give trainees hands-on experience obtaining anatomical ultrasound images necessary for regional anesthesia blocks in a guided-virtual setting and to evaluate the effectiveness of skills acquisition. This workshop included two pediatric anesthesiology fellows and a pediatric anesthesiologist.

Trainees were instructed on ultrasound-guided regional block acquisition. For two weeks, trainees acquired images/movies of regional block anatomy at home using their own children. Virtual video assistance was available. Trainees then used acquired images/movies to discuss needle and local anesthetic placement with a pediatric regional anesthesiologist. Trainees completed pre- and post-workshop surveys assessing attitudes, perceived educational efficacy, and procedural skill acquisition. The faculty member also assessed trainees’ skills.

The virtual workshop was successful. Trainees expressed successful active learning and increased comfort in performing regional blocks on live patients. They correctly identified relevant anatomy of acquired images/movies, as well as needle and local anesthetic placement at the time of debriefing. Faculty were pleased with trainees’ initial performance of regional blocks.

Adapting an in-person workshop to an at-home guided experience is a safe, feasible, and well-received method for anesthesiology trainees to obtain experiential learning of ultrasound-guided regional anesthesia. This facilitated at-home learning experience allows for hands-on skill practice while preventing exposure of child models to the hospital setting during a pandemic.

## Introduction

Regional anesthesia has become a central component of pediatric anesthesiology (PA) practice and training [1], and ultrasound-guided regional anesthesia is now the standard of care for the majority of regional blocks [2]. PA fellowship programs must teach trainees to safely and efficiently perform ultrasound-guided regional blocks (UGRBs) for intra- and post-operative pain control. To produce true competency, a sufficient quantity of UGRBs must be paired with high-quality assessment and constructive feedback [3,4]. Hands-on learning with simulation and live models has been the gold standard for education, allowing trainees to combine knowledge from texts and hands-on skills [5-7].

Experiential learning is critical to solidify new skill sets. Simulation engages the adult learner to be actively involved in the process of medical learning. A systematic review by Chen et al. (2017), assessing ultrasound-guided regional anesthesia simulation training, found that simulation training significantly improved UGRB knowledge and skills compared to non-simulation (i.e., didactic) teaching [8]. Coronavirus disease 2019 (COVID-19) has altered our ability to safely hold in-person, live model workshops. Like other novel programs pushing technological frontiers, we must continue to adapt experiential learning so that trainees do not miss out on beneficial educational opportunities [9,10]. We piloted a new curriculum for an at-home, guided virtual workshop using a handheld portable ultrasound. The trainees utilized family members and their children (when appropriate and able) to practice pediatric UGRBs. We present a technical report of our pilot workshop and a preliminary analysis of our experience.

## Technical report

The purpose of this virtual guided workshop was to develop an effective experiential learning program for ultrasound acquisition skills without the need for live child models. The main goal was to give trainees hands-on experience obtaining anatomical ultrasound images necessary for regional anesthesia blocks in a guided virtual setting and to evaluate the effectiveness of skills acquisition. Qualitative thoughts on effective use of personal time management were also elicited.

**Figure 1:**
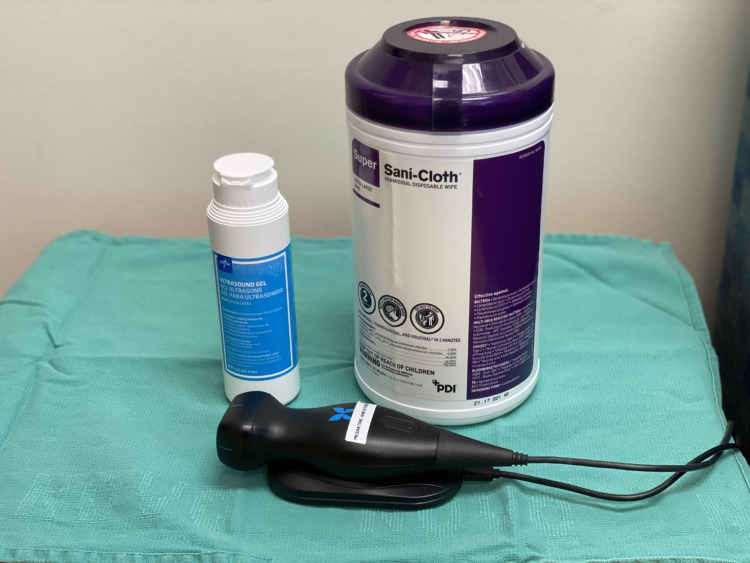
Supplies for home imaging.

We surveyed our 2020 PA trainees to ascertain interest in this type of at-home workshop, experiential training history, and self-evaluation of UGRB knowledge (Table 1). Trainees then met individually with a pediatric regional anesthesiologist to briefly preview how to image 12 different UGRBs commonly used in anesthetic management (Table 2). In addition, they were shown how to download the Butterfly iQ-Ultrasound app (Butterfly Network, Guilford, Connecticut) on their devices and to use the Butterfly iQ+ probe provided by the department. Other portable ultrasound devices are available, but we chose the Butterfly iQ+ as it was available for use at our institution. A “quick tips” guide for UGRB acquisition was provided. We instructed trainees to use their children to obtain images/movies of the aforementioned UGRBs over a two-week period and save their self-selected best images for evaluation. Trainees took home ultrasound gel, cleaning solution, and a Butterfly iQ+ ultrasound probe for use on their smart devices (Figure 1). We provided each trainee the option of participating in individual, one-hour video conferencing sessions with a pediatric regional anesthesiologist to guide them while acquiring images at home.

**Table 1:** Pre-workshop assessment results. a. Rated on a five-point scale (1 = not comfortable performing at all; 3 = know how to perform but need regular redirection; 5 = require no assistance). b. Rated on a five-point scale (1 = never used; 3 = have used for regional block and vascular access, still require some direction; 5 = extensive/expert level).

Question	Trainee 1	Trainee 2
What is your comfort level in performing regional blocks?^a^	5	4
What is your comfort level in performing pediatric regional blocks?^a^	5	3
Have you ever participated in a live model regional workshop before?	Yes	No
If you have participated in a live model regional workshop, did you find it helpful to learn regional anesthesia?	Yes	N/A
Have you had ultrasound experience prior?^b^	5	3
Are you comfortable taking a cleaned ultrasound probe home to image your child?	Yes	Yes
Do you have the ability to download the Butterfly^TM^ app?	Yes	Yes
What are your goals for this workshop/activity?	Gain facility with a new ultrasound system and assess its effectiveness when considering a possible purchase for future mission trips, and show my child his insides.	Become more familiar, proficient, and efficient with various pediatric regional blocks, especially blocks used in the "real world." Also figuring out which blocks for which surgeries, proper positioning, appropriate dosing, etc.

**Table 2:** Regional blocks to be captured.

Upper extremity	Truncal	Lower extremity
Interscalene	Rectus sheath	Femoral
Supraclavicular	Transversus abdominis plane	Popliteal
Infraclavicular	Quadratus lumborum	Saphenous
	Erector spinae plane	
	Lumbar plexus	
	Paravertebral	

At the conclusion of each two-week period, a pediatric regional anesthesiologist met with each trainee individually. Trainees presented their self-selected best images/movies of each assigned UGRB. Trainees described anatomy, projected needle placement and trajectory, and location of local anesthetic deposition as well as dosing. The anesthesiologist and trainee discussed which surgeries each block could be used for, highlighting those most commonly performed at our training hospital. In addition, they discussed questions and challenges that arose while performing the UGRBs at home.

In addition to the debriefing, the pediatric anesthesiologist formally evaluated each trainee’s images in regard to anatomy recognition, local anesthetic deposition site, and needle insertion site. Evaluations were incorporated into the quarterly milestone assessments for each trainee. As part of continuous curriculum refinement, trainees completed a seven-question post-workshop assessment evaluating the self-perceived effectiveness and desirability of the new workshop format (Table 3). Members of the pediatric acute pain service completed evaluations of trainee performance during their rotations, gauging satisfaction with both workshop and subsequent trainee performance.

**Table 3:** Post-workshop assessment results. a. Rated on a five-point scale (1 = much less efficient with personal time; 3 = neutral; 5 = much more conducive to personal time/individual learning).

Question	Trainee 1	Trainee 2
Did you find the workshop taught you pediatric regional block anatomy?	I found it to be a good way to assess block anatomy in my own time, without time pressure.	Yes, definitely helpful to see the anatomy and practice getting the views.
Compared to other group workshops, did you feel this format facilitated learning?	Yes	Absolutely. Capturing video snippets of each block afforded more ownership and incentive to properly achieve them than just a quick hands-on turn at a workshop.
Did you feel safe having your child/children participate?	Yes, I loved that part.	Yes
Were you able to successfully get your child to participate?	Yes	Yes
How did you find this format respected your personal time versus a traditional workshop?^a^	5	4. Took me a decent amount of time because I was trying to teach myself some views I'd never done or seen done in real life before; otherwise would have gone more quickly.
If offered the choice in the future, would you prefer this format or a traditional two-hour live model group workshop? Why?	I think this approach has advantages, but there is a distinct advantage to having an expert looking over the shoulder of the learner and advising them in real time. I would likely choose the live model group workshop for this reason. This was perfect given the pandemic circumstances.	I would still enjoy a group workshop for in-person feedback on achieving good block images particularly for the blocks I hadn't done before (i.e., lumbar plexus, paravertebral), but would also be interested in pairing that with bringing home the probe for opportunity on our own time to practice getting the images.
Additional comments?	N/A	Definitely a valuable educational tool, especially in the time of a pandemic, but even if not!

## Discussion

The purpose of this technical report was to provide readers, educators, and physicians who train residents and fellows in ultrasound-guided regional anesthesia a framework for a successful, virtually guided UGRB workshop. Based on our experience, this workshop format was both well-received and educational. Trainees highly rated the format in that it provided more time to handle the ultrasound, more easily fit their personal schedules, and allowed them to reference multiple aids (books, online modules, and video conferencing) throughout. The creation of personal images for future reference throughout their training/career was felt beneficial.

Anesthesiology residents are becoming increasingly exposed to adult regional anesthesia during training [11]. However, their exposure to pediatric regional anesthesia remains low. Experiential learning is necessary to optimally teach procedural skills. Given the ever-changing environment of the world, educators must adapt educational experiences to both meet educational needs and protect all involved. At our institution, this virtual workshop adaptation achieved both of these goals and was well-received by all.

Even in the absence of a global pandemic, this type of at-home workshop conveys distinct benefits. Participants noted appreciation for being able to include their families in their studies, and their children expressed excitement at being included in their parent’s work. In informal discussions with trainees, they noted a higher level of comfort in their own children than that observed in our prior practice of bringing faculty’s children into the hospital for a large group hands-on workshop. As children were examined in their homes by their own parents, they were more at ease and participated more. In the spirit of adult learning, trainees could take ownership of this learning activity on their own time. Furthermore, banked files of images/movies from trainee experiences will offer more material to draw from, helping them in their future careers.

Given the extended time to complete, trainees could repeat scans multiple times to create their final image portfolio. Trainees came to the debrief prepared to present and ask more questions than typically encountered in a two-hour, live-model workshop. Additionally, the trainees had an intimate relationship with their individual models, enabling image acquisition with minimal discomfort.

A study by Udani et al. (2017) assessed the comparative effectiveness of simulation-based deliberate practice versus self-guided practice on novice anesthesiology residents’ acquisition of UGRB skills, finding no difference in acquisition and retention of skills. However, they did find that self-guided practice required less time and faculty resources [12]. Our adaptation of an in-person workshop to an at-home workshop that still utilizes a live model is perhaps the perfect hybrid of simulation-based and self-guided practice.

Potential future directions include the utilization of acquired trainee images in future lectures and workshops to help educate others. While piloted with pediatric anesthesiology fellows, it could easily be expanded to resident physicians. The addition of pre- and post-knowledge tests would better ascertain knowledge acquisition.

Limitations include the possibility that every trainee may not have appropriately aged children in their lives. Further, even among trainees that do have children, the children may not adequately participate. In these cases, the learners could obtain ultrasound images on themselves, or other immediate or extended family members regardless of age. Not all training programs have access to portable ultrasound probes and the requisite devices to utilize them; this could be alleviated through educational grant funding. Lastly, the instructor is not present 100% of the time to offer guidance, though individual times can be set up for additional help.

## Conclusions

This virtual workshop adaptation allowed for a successful continuation of trainee education on fundamentals in pediatric UGRB with live models, without further physical exposure of additional physicians or children. With the continued presence of the COVID-19 pandemic, we should strive to educate our trainees in the safest environment possible. This facilitated at-home workshop allows for hands-on skill practice while preventing exposure of child models to the hospital setting. Given the benefits across multiple domains for both trainees and faculty, we plan to offer pediatric UGRB workshops in this manner.
